# Movement and ranging patterns of the Common Chaffinch in heterogeneous forest landscapes

**DOI:** 10.7717/peerj.368

**Published:** 2014-06-19

**Authors:** Katrin Kubiczek, Swen C. Renner, Stefan M. Böhm, Elisabeth K.V. Kalko, Konstans Wells

**Affiliations:** 1 Institute of Experimental Ecology, University of Ulm, Ulm, Germany; 2 Smithsonian Conservation Biology Institute at the National Zoological Park, VA, USA; 3 The Environment Institute, School of Earth and Environmental Sciences, The University of Adelaide, Adelaide, Australia

**Keywords:** Animal tracking, Bird movement capacity, Habitat use, Landscape heterogeneity, Hierarchical habitat selection, Multilevel hierarchical regression

## Abstract

The partitioning of production forests into discretely managed forest stands confronts animals with diversity in forest attributes at scales from point-level tree assemblages to distinct forest patches and range-level forest cover. We have investigated the movement and ranging patterns of male Common Chaffinches, *Fringilla coelebs*, in heterogeneous forest production landscapes during spring and summer in south-western Germany. We radio-tracked a total of 15 adult males, each for up to six days, recording locations at 10-min intervals. We then performed point-level tree surveys at all tracking locations and classified forest stand attributes for the areal covering of birds’ ranges. Movement distances were shortest in beech forest stands and longer in spruce-mixed and non-spruce conifer stands. Movement distances increased with stand age in beech stands but not in others, an effect that was only detectable in a multilevel hierarchical model. We found negligible effects of point-level tree assemblages and temperature on movement distances. Daily range estimates were from 0.01 to 8.0 hectare (median of 0.86 ha) with no evident impact of forest attributes on ranging patterns but considerable intra-individual variation in range sizes over consecutive days. Most daily ranges covered more than one forest stand type. Our results show that forest management impacts the movement behaviour of chaffinches in heterogeneous production forest. Although point-level effects of movement distances are weak compared with stand-level effects in this study, the hierarchical organization of forest is an important aspect to consider when analysing fine-scale movement and might exert more differentiated effects on bird species that are more sensitive to habitat changes than the chaffinch.

## Introduction

The movement of an animal is an intricate interplay of its life history, behaviour, and response to the underlying habitat (Morales & Ellner, 2002; Morris, 2003; Moorcroft, Lewis & Crabtree, 2006; Schick et al., 2008). Movement through space to acquire essential resource is the basis for long-term survival and reproduction. In turn, the way that animals move and use environmental space have an impact on interactions with other organisms or resources and thus affect ecosystem processes, such as when and where prey species are consumed (Van Bael, Brawn & Robinson, 2003; Böhm, Wells & Kalko, 2011) or where defecated seeds are dispersed, thereby playing a role in forest succession and functioning (Breitbach et al., 2012). The investigation of movement is therefore important for understanding the relationships of animals with their biotic and abiotic environment, and, in turn, the way that environmental changes possibly affect animals from individual movement to population and community structure (Ovaskainen, 2004; Kokko & López-Sepulcre, 2006; Morales et al., 2010). On this background, an understanding of whether movement and space use differs in structurally homogeneous and heterogeneous environments might provide us with important information about the behaviour of an animal and its potential for adaptation to changing environmental conditions (Zollner & Lima, 1999; Morales et al., 2004; Forester et al., 2007). Moreover, if environmental heterogeneity is an issue at different scales such as landscapes, habitat patches, and point locations where animals move, identifying which scale is most influential on movement and behaviour may aid in addressing the appropriate scale for management action in informed conservation efforts.

The temperate production forests of Central Europe are commonly managed as discrete forests stands of limited sizes and many mobile organisms such as birds are likely to encounter various forest patches within their ranges (Andrén, 1994; Wells et al., 2011). Studies on the way in which local environmental conditions affect fine-scale movement and habitat selection in heterogeneous landscapes emphasize that movements are often driven by combinations of environmental attributes from different scales, in addition to individual factors of the animals (Rettie & Messier, 2000; Johnson et al., 2002; Dalziel, Morales & Fryxell, 2008; Leblond, Dussault & Ouellet, 2010). Moreover, foraging and habitat selection are known to be hierarchical, with fine-scale patch selection taking place within different landscape units, which, in turn, might be subject to selection at larger scale (Kotliar & Wiens, 1990; Fauchald, 1999; Rolstad, Løken & Rolstad, 2000). Within forest landscapes, for example, bird abundance might increase within patches of certain tree assemblages (Lee et al., 2002). Analytical tools such as mixed effect and state-space approaches are increasingly being considered in the investigation of fine-scale spatiotemporal patterns in movements and the translation of natural hierarchical processes into corresponding models (Wikle, 2003; Fieberg et al., 2008).

Here, we studied the movement and ranging of a generalist and omnivore passerine species, the Common Chaffinch, *Fringilla coelebs*, which is known to utilize a large range of forest types and open habitats, feeding on arthropods and seeds, and to exhibit territorial behaviour (Glutz von Blotzheim & Bauer, 1997; Hanski & Haila, 1988; Hanski, 1992; Maciejok, Saur & Bergmann, 1995; Whittingham et al., 2001). Despite its low specialization in habitat use, we expected the movement and ranging patterns of this species to differ among individuals that inhabit diverse forest types, as forest management has an impact on a large variety of species and thus on potential resources and habitat conditions (Paillet et al., 2010). We further expected that fine-scale forest composition and structure, i.e., the types and density of trees around foraging locations, would have a similar or greater impact on movement trajectories as larger-scale forest cover with various tree stands arranged as adjacent patches, since we suspected that such fine-scale environmental conditions would be of more relevance to immediate foraging and movement decisions than overall forest characteristics at the landscape scale. If local tree assemblages provide many resources because of high tree diversity and density, for example, we anticipated relatively small movement distances, as birds should be less motivated to move to distant locations. Thus, we expected longer movement distances in generally unsuitable than in suitable habitat. Likewise, if movement distances are shorter in favorable forest types, we expect daily ranges to be smaller.

**Figure 1:**
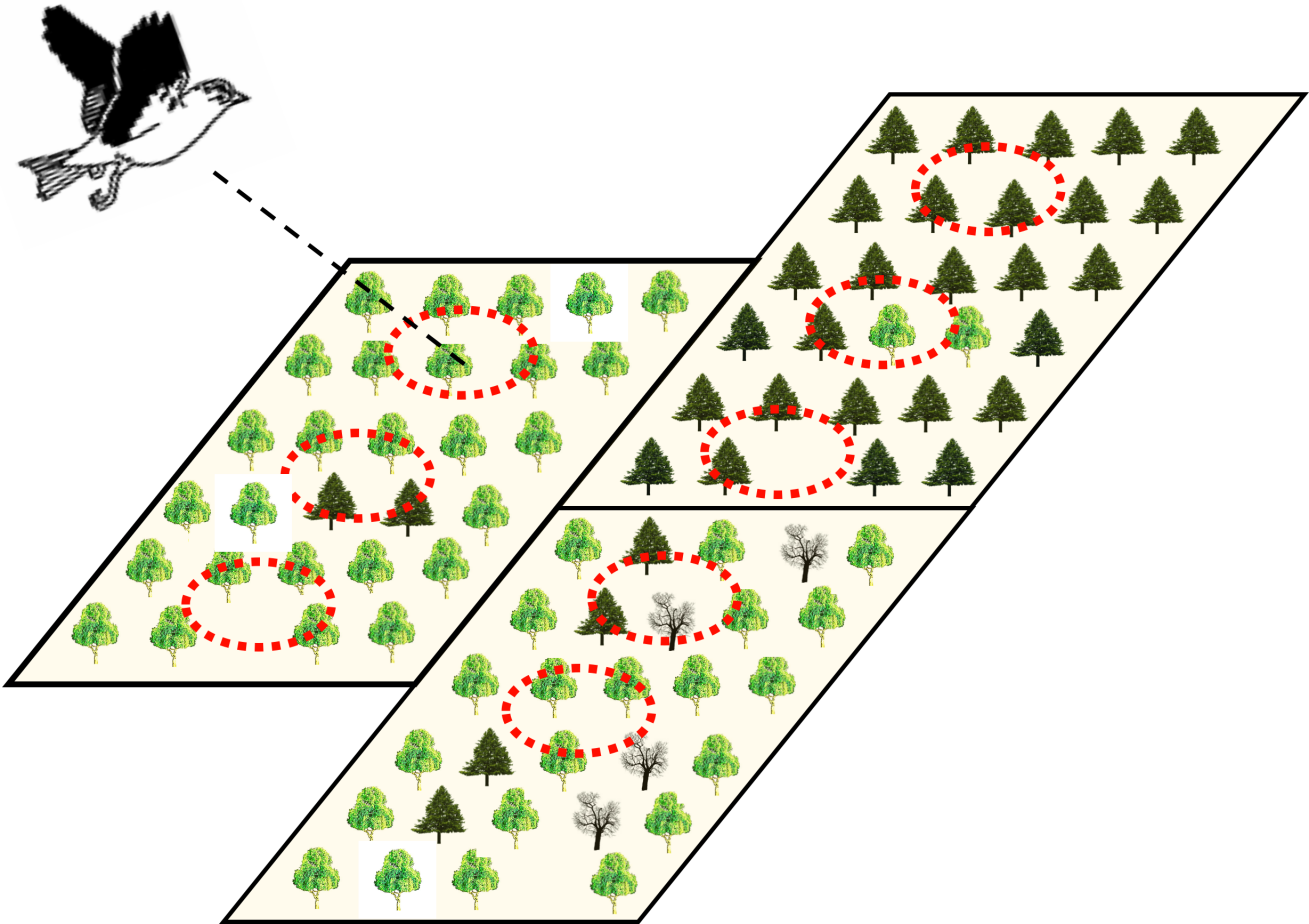
Illustration of the potential hierarchical organization of point- and stand-level forest attributes hypothesized to be influential on bird movement and ranging in heterogeneous production forest landscapes. Large rectangles represent three adjacent forest stands with different management practices and dominance of different tree species (represented by three different tree symbols). Within forest stands, local tree assemblages might differ in their composition and tree density, such that point locations (dashed-line circles) may be as different within as among the different forest stands.

We further aimed to discuss the hierarchical structure of forest attributes. Linked to our working hypothesis above, we assumed movement distances to differ among forest stand types (i.e., forest stand types differ in their habitat suitability and should thus predict differences in movement) and, concomitantly, point-level effects of tree assemblages on movement distances to be nested within different forest stands. For conceptualizing the natural hierarchy into a corresponding analytical framework, we therefore considered multilevel analytical frameworks to be of particular relevance in heterogeneous and patchy production forests in comprehensive tests of the way that nature drives movement trajectories (Fig. 1).

## Materials and Methods

### Study area and bird capture

Our study was conducted in south-western Germany in the Schwäbische Alb (approximate central coordinates: 48.41°N, 9.50°E, elevations of 500–800 m a. s. l.), a montane environment with a long history of anthropogenic forest management and small-scale agriculture. The land cover in the area was originally dominated by beech forest (*Fagus sylvatica*) but has been significantly altered by the logging of some of the forests and a variety of farming practices (Fischer et al., 2010). The forests are characterized by relatively small and discrete woodland stands with distinct forest management strategies (mean area of the forest stands 3.6 ± 5.8 ha; min/max: 0.004/84.5 ha; *N* = 1549) and interspersed open habitat. Forest stands range from old beech stands, neglected by management for more than 100 years, to mixed-deciduous/coniferous forests with limited intensive logging to intensively farmed monocultures of beech or Norway spruce (*Picea abies*). Each forest type is represented by a range of age classes from young plantations to stands with trees of up to 140 years old.

We captured and marked a total of 15 male adult chaffinches, *Fringilla coelebs*, in the field between May and July 2009 (5 individuals) and between April and June 2010 (10 individuals, one bird tracked in 2009 was recaptured and also tracked in 2010). During this time, most males had well-defined territories, which they defended against other males that usually stayed in close proximity to each other in the study area (K Kubiczek, pers. obs., 2010). Birds were captured in the vicinity of so-called experimental plots of the research platform ‘Biodiversity Exploratories’, of which each was characterized by homogeneous forest cover of at least 100 ×100 m (http: //www.biodiversity-exploratories.de; Fischer et al., 2010). A large range of abiotic and biotic information are collected at these plots, including temperature measures; however, we avoided trapping birds directly there in order to avoid disturbing other experiments and, also, as bird ranges cannot be directly linked to a plot-based monitoring scheme. We captured birds with nylon mist nets placed in front of playback units. We measured wing size as the length of the third primary with a pinned metal ruler to the nearest millimetre and body mass with a spring balance to the nearest 100 g/1 g division (Pesola, Baar, Switzerland).

Each captured bird was ringed with a uniquely numbered leg ring (Vogelwarte Radolfzell, Germany). For radio tracking, birds were equipped with a small radio transmitter (BD-2G, Holohil Systems Ltd., Ontario, Canada), which weighed 1.2 g and was therefore within the 3–4% body mass range assumed suitable for carrying without causing any harm to or having any impact on the behaviour of small passerine birds (Withworth et al., 2007). We constructed a backpack harness out of an elastic plastic band (High Power Gum, 0.6 mm, Lenzi) to which the transmitter was attached with a small drop of adhesive glue (Loctite, Henkel, Düsseldorf, Germany). The harness fixed the transmitter on the back of birds with loops around the legs; details of such bird harnesses have been described in (Naef-Daenzer, 2007). After checking for the accurate fit of the harness, we immediately released the bird at the point of capture.

Capturing and handling birds were performed in compliance with federal and state laws. All permits were granted by the “*Regierungspräsidium Tübingen, Referat Artenschutz, Tierschutz*” (*RPT Tierversuch-Nr. -884, -907, -1056*). All birds were handled to best practice following the guidelines of the bird banding laboratory “*Vogelwarte Radolfzell*” and we minimized suffering through swift release into the wild and professional harnessing techniques as recommended by the permit authorities. These guidelines on bird handling for scientific purpose implemented all steps requested by the animal welfare of the European Commission, which are implemented in the federal and state laws of Germany.

The mandatory training of the field workers was assessed during the permit procedure. Access to land was approved by all land owners.

### Bird tracking

We used VHF radio-telemetry with a hand-held Yaesu VR-500 receiver (Yaesu Musen Co. Ltd., Tokyo, Japan) combined with a modified H-aerial antenna (Wagener Telemetrieanlagen, Köln, Germany) for tracking birds at regular time intervals with the so-called homing-in method. During tracking, we narrowed down the birds’ location by a single observer repeatedly checking the direction of the intensity of the radio signal from different angles. Birds usually perched in the canopy of larger trees, about 5–20 m above ground, and approaching these trees, we were able to estimate the geographic locations with a precision of ca. 10 m. With this method, we recorded bird locations in regular 10-min intervals. Only occasionally birds moved too large distances to follow them and accurately determine their location in 10-min intervals (about 20 times in >2,000 location records).

Each bird location was marked with a ribbon attached to the nearest branch or tree and an individual location number and we noted the time of observations. During tracking, we estimated coarse geographic position with a conventional handheld GPS with relatively large error of ca. 10–15 m (Garmin GPSmap 62CX, USA) for orientation only, whereas we determined the exact geographic coordinates of marked locations with a Trimble geoXTTM GPS and GeoBeaconTM receiver (Trimble, USA) with an accuracy of <1 m some days after radio tracking was finished.

We collected a first set of radio-telemetry data for each individual two days after the radio transmitter was attached to the bird, tracking birds from 10–30 min prior to sunrise for a minimum of 6 h and at a minimum of 30 accurate tracking locations at 10-min intervals. We radio-tracked individuals on three consecutive mornings. Another set of tracking was conducted after a break of 3–7 days, repeating the same protocol and gathering information for another three days. As we expected to cover the main daily activity period of birds with this tracking, we refer to ‘daily ranges’, being aware that this is only a relative measure that should not be confused with absolute home-range estimates.

### Environmental characterization

At each bird location, we estimated local tree density and species composition by using a so-called dendrometer. For this, a metal bar of 15 mm width was held in a constant distance of 50 cm from the observer’s eye at the bird locations. All trees with diameters at breast heights ⩾15 cm and encountered as exceeding the width of the metal bar were counted. These trees were identified with regard to their species and diameter and their distance to the bird location were recorded. This method is used in forestry to estimate tree stock (Kramer & Akca, 1987) and proved to be an effective measure of local tree assemblages in the vicinity of bird locations.

For classifying area-wide forest cover and the forest stand types that birds used, we used a digital regional forest management map, which had information on tree species composition, stand age and management plan on the stand-level of discrete management units and which was obtained from the regional authorities (‘Forsteinrichtungsdaten’; ‘Fogis 2006’, Regierungspräsidium Tübingen). The digital maps accurately described at least 90% of forest stands, as confirmed by our own field inventories. We categorized forest stand composition (based on beech and spruce as the dominant overstory tree species, with a threshold level of 70% of dominant trees) into six categories: beech, beech-mixed, deciduous-mixed, spruce, spruce-mixed and coniferous-mixed (Wells et al., 2011). Stand age was defined as the oldest tree layer within stands. Forest management classes included age-class forest and unmanaged forests.

Local temperature data were obtained from meteorological measurement stations at nearby experimental plots (Rotronic HygroClip S3 Air Temperature sensor) in the vicinity of tracking locations and were measured at heights of 2 m at 60-min-intervals (we assigned the measured temperature value to all bird locations until the next measurement was available).

### Modelling approach and data analysis

We performed data analysis at two different levels, namely at point-level movement distances (distance moved per 10-min time intervals) and the level of daily ranges (areal metric derived from subsets of points). Whereas these data sets are linked, in that ranges are estimated from subset of point locations, we point out that environmental attributes need different consideration at these two scales. In general, any point location used by a bird at a particular time can be described by local tree assemblages at this location nested within the forest stand in which this point is located (e.g., we can expect two point locations in a beech forest to differ in local tree assemblages if mixed with other tree species or if spatial clumping results in different tree densities; likewise, if two points with the same point-level attributes are located in a different forest stand, we can expect this to be a different environment, see Fig. 1). Each point location used at a particular time is further characterized by the local temperature and daytime. In contrast, at the range level, we can characterize the forest environment that has been used by a bird during a certain time as the average of point-levels attributes from utilized points. Independently of point estimates, we can characterize the overall forest landscape utilized during a certain time period from area-wide forest maps by describing the forest landscape underlying the resulting range. For a better understanding, we superscript environmental variables below with ‘^*P*^’ for point-level, ‘^*S*^’ for stand-level and ‘^*R*^’ for range level attributes (see Table 1 for a detailed description of environmental variables).

**Table 1:** Description of environmental covariates at range and point-level used to examine possible predictors of variation in daily range size and movement rates of birds in various forest stands.

Variable	Description	Value mean/median and range
**Point-level**		
*B^P^*	Number of beech trees (diameter at breast height *DBH* >15 cm) around tracking location	3.1 (0–29)
*S^P^*	Number of spruce trees (*DBH* >15 cm) around tracking location	11.7 (0–42)
*X^P^*	Number of all trees (*DBH* >15 cm) around tracking location	16.4 (0–42)
*H^P^*	Shannon–Wiener index of tree species diversity (*DBH* >15 cm) around tracking location (based on *H*′ = −∑*p_s_*log(*p_s_*), where *p_s_* is the proportional abundance of species *s*)	0.4 (0–1.8)
*T^P^*	Local temperature at tracking location during time of observation	9.5 (−1.7–21.5) °C
*Dt^P^*	Time difference of observation to sunrise time of the same day	169.8 (−30–496) min
*S.type^S^*	Type of forest stand surrounding tracking location based on dominant tree species as classified by regional forest management map	5 different levels (Beech, Beech-mixed, Spruce, Spruce-mixed, Non-spruce conifer)
*S.age^S^*	Age of forest stand surrounding tracking location as classified by regional forest management map	69.4 (0–140)
**Range-level**		
*JulDay^R^*	Day of observation counted onwards from 1st March, comprising a continuous seasonal measure; foliage density and arthropod species presence can be expected to change because of the strong seasonal variation in the study region	54 (9–111)
*Temp^R^*	Mean temperature [C] during the time of daily tracking (averaged from point-level measures)	8.3 (0.8–17.9)
*StandNo^R^*	Number of distinct forest stands, with distinct forest management practice and tree types; derived from regional management map	2 (1–8)
*BeechStand^R^*	Proportion of area underlying the daily range estimate covered with forest stands dominated by beech; derived from regional management map	16 (0–100)
*SpruceStand^R^*	Proportion of area underlying the daily range estimate covered with forest stands dominated by spruce; derived from regional management map	47 (0–100)
*BeechTree^R^*	Average number of beech trees at point locations within daily range; averaged from 30 locations used for daily range estimate	1.3 (0–17.0)
*SpruceTree^R^*	Average number of spruce trees at point locations within daily range; averaged from 30 locations used for daily range estimate	11.7 (0–28.8)
*TotalTree^R^*	Total average number of trees at point locations within daily range; averaged from 30 locations used for daily range estimate	18.2 (1.0–30.4)

We calculated movement distance as the bee-line distance between bird locations estimated at consecutive time steps of 10 min. For this, we considered all records with time lags between 8 and 12 min, as field conditions do not allow to sample in strictly regular time intervals. For analysing possible relationship between movement distances and environmental conditions, we matched covariates with the starting point from which movement distance had been calculated. We found point-level characterization of the forest at GPS locations more feasible than characterizing habitat along straight-line sections between points (see Fortin et al., 2005 for an example of step-selection functions) and also on the rationale that habitat along lines can be assumed to be less influential during flight. We constructed a hierarchical model, so that we were able to translate the hierarchical structure of points nested in different forest stand types into an analytical framework. Each observed movement distance *λ*(*i*, *d*, *t*) of bird individual *i* at observation day *d* and time *t* was characterized by the point-level number of beech trees *B^P^*, the number of spruce trees *S^P^*, the local density of all tree species *X^P^* and the Shannon diversity index of all tree species *H^P^* at respective point locations. *T^P^* was the ambient temperature assigned to the respective location and time and we noted daytime *Dt^P^* as the minute time difference of observations to sunrise time. Each point location was characterized by its surrounding forest stand type *S*.*type^S^* and stand age *S*.*age^S^*. We assumed that the effects of point-level conditions (variables with ‘^*P*^’) were not independent of the surrounding forest stand type. Likewise, the effect of stand age could be assumed to vary with stand type.

We further assumed the movement distance to exhibit some autocorrelation in space and time because of similar environmental conditions or intrinsic factors such as the motivation of a bird to move, so that *λ*(*i*, *d*, *t*) might depend on *λ*(*i*, *d*, *t*−1), which is a 1st order autoregression term. Modelling movement distance at the log-scale with these assumptions gives the following model:

\begin{eqnarray*} \log \left(\lambda (i,d,t)\right)\sim {\mu }_{0}(i)+\alpha (i)\lambda (i,d,t-1)+S.t y p{e}^{S}+{\beta }_{1}(S.t y p{e}^{S}){B}^{P}+{\beta }_{2}(S.t y p{e}^{S}){S}^{P}+{\beta }_{3}(S.t y p{e}^{S}){X}^{P}+{\beta }_{4}(S.t y p{e}^{S}){H}^{P}+{\beta }_{5}(S.t y p{e}^{S}){T}^{P}+{\beta }_{6}(S.t y p{e}^{S})D{t}^{P}+{\beta }_{7}(S.t y p{e}^{S})S.a g{e}^{S}+\varepsilon . \end{eqnarray*}

Here, *μ*_0_(*i*) is the intercept that is allowed to vary among individuals. Nested/hierarchical structure of point-level attributes are implemented via coefficient estimates *β*, which are allowed to vary for different forest stand types; stand type is thus considered as both a group-level predictor and a grouping indicator. The zero-mean Gaussian error ε captures both variation due to observation error and residual variance of the process model, as our model framework did not explicitly account for measurement errors in a separate observation model. We did not consider variable selection in our modelling efforts, as we were more interested in inferring the strength and different magnitudes in coefficient estimates, rather than model parsimony.

We fitted the model with a Bayesian approach by using Gibbs sampler as implemented in the OpenBUGS software (Lunn et al., 2009) with the vague uniform priors of U(0, 100) assumed for all variance terms. All covariates were scaled to a mean of zero and one SD prior to analysis. There was no collinearity between covariates.

We ran two MCMC chains and found convergence and stationary posterior distribution after visual inspection of 100,000 iterations, which were discarded. Posteriors were estimated from 5,000 further iterations for each chain as the highest posterior density mode and 95% credible intervals (CI). Covariate relationships were assumed to be statistically significant if 95% CI did not overlap zero. We estimated finite sample variances for estimating the % variance explained in the data by different predictors in our model (see Appendix S1 for model code and further information).

For estimating the daily ranging patterns of birds, we calculated Convex hull polygons for each days’ range estimate (Getz & Wilmers, 2004), by using the first 30 tracking points per day. Daily ranges were then estimated with the fixed nearest neighbour method with *k* = 12 points and areal range sizes were estimated as 90% isopleths. Range analysis were conducted with the LoCoH.k() function of the adehabitatHR package in R (R Development Core Team, 2013).

For examining possible relationships between range size and environmental covariates, we used a linear mixed effects model fitted with a restricted maximum likelihood approach (using the lmer() function in the lme4 package in R) to account for the repeated measure of ranges from the same individual as a grouping structure, since our longitudinal set of ranges comprised variation in range sizes over time for any individuals and variation in range sizes among individuals under various environmental conditions. As a full model, we modelled daily range size at log-scale by using individual bird identity *Bird^R^*, mean temperature *Temp^R^*, julian day *JulDay^R^*, stand numbers *StandNo^R^*, proportion of beech stands *BeechStand^R^*, proportion of spruce stands *SpruceStand^R^* underlying the ranges and the average number of beech trees *BeechTree^R^*, average number of spruce trees *SpruceTree^R^* and total average number of tree species *TotalTree^R^* per location within daily ranging as covariates (see Table 1). For the latter six covariates describing forest attributes within daily ranges, we introduced bird identity as random effects. We constructed various models with only single covariates and subsets and compared model outputs visually and based on Akaike’s information criterion and the size and variation in coefficient estimates (as none of the covariates proved to be of relevance, details not outlined).

## Results

We radio-tracked 15 male chaffinches in various forest types, of which eleven were tracked twice for three consecutive days (one bird was tracked in two years, for which results are displayed as different individuals) and five were tracked once for three consecutive days (providing a total of 81 daily ranges). This resulted in a total of 2,316 movement distances at 10-min intervals for analysing environmental and individual impact on movement distance.

### Movement distances

**Figure 2:**
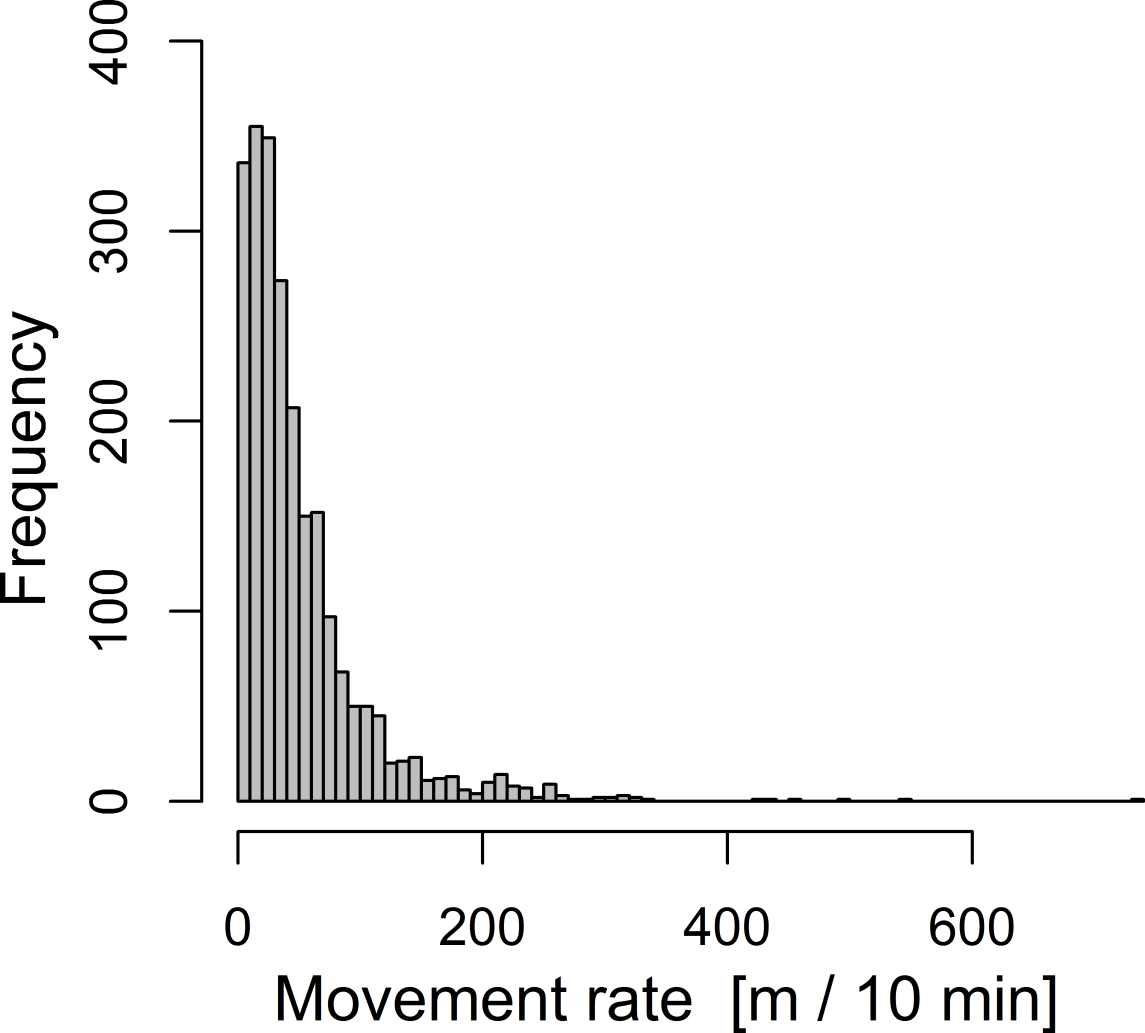
Overall frequency distribution of movement distances of 16 radio-tracked chaffinches during 10-minute intervals. The total sample size is 2,316 distances. Note that the relative frequency distribution of short and long movements is not necessarily equal among individuals and is here only pooled for illustration.

Movement distances exhibited a highly skewed distribution towards small movements within 10-min intervals and only occasional large movements (Fig. 2). In 117 out of 2,316 records (5%), birds did not move but perched at the same location for at least 10 min. Median movement distance was 35 m during the 10-min intervals and the longest recorded movement was 730 m (note that a few longer movements were likely to be missed due to difficulties in following long distances during tracking).

**Figure 3:**
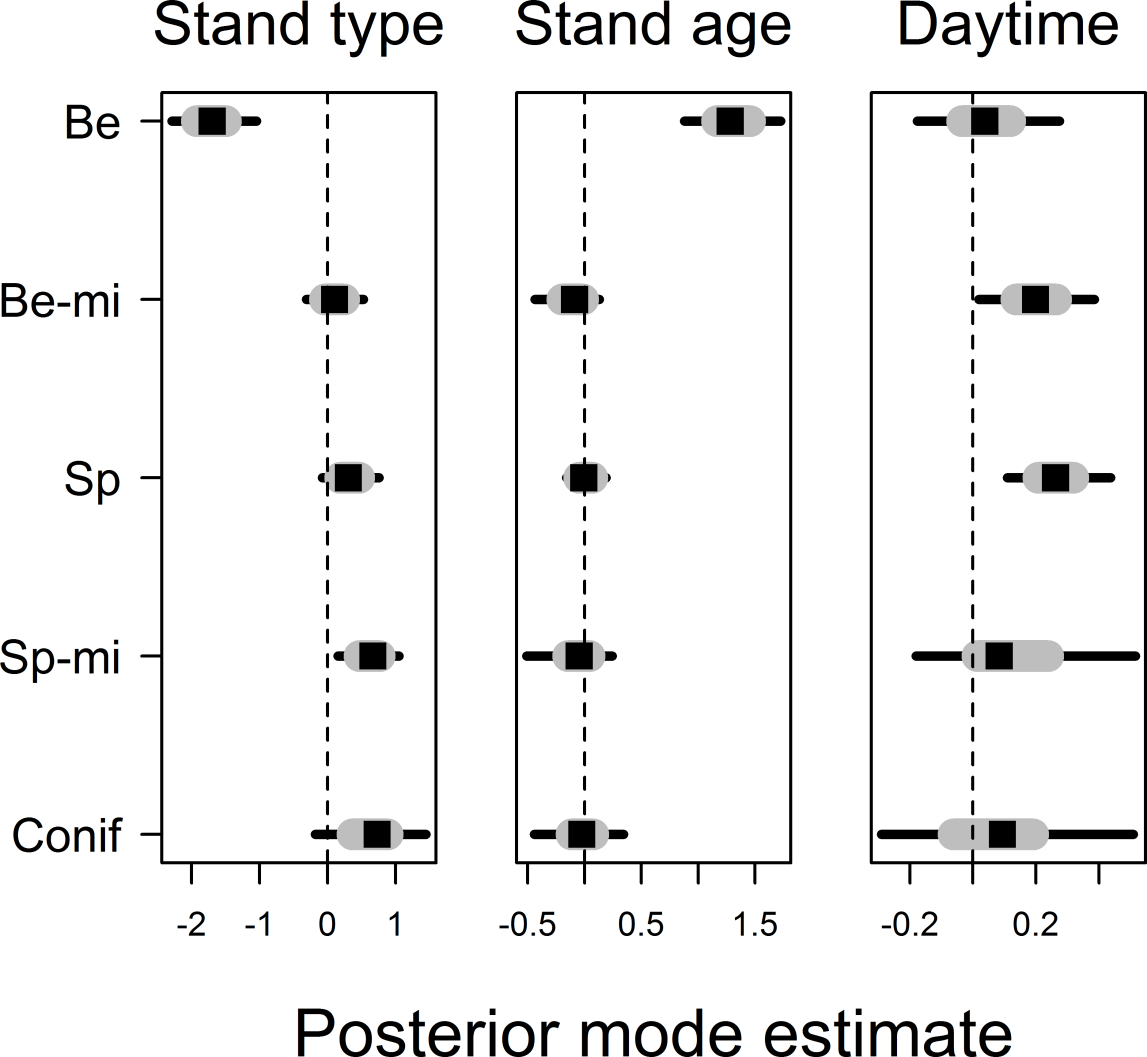
Posterior coefficient estimates for the effects of forest stand type and the effects of stand age and daytime on movement distance. Note that stand age and daytime effects are nested within different stand types and estimates were allowed to vary among them. Stand types are given as Be, Beech; Be-mi, Beech-mixed; Sp, Spruce; Sp-mi, Spruce-mixed; Conif, Non-spruce conifer. Credible intervals are drawn as grey bars for 50% and black bars for 95% intervals.

Movement distances differed among forest stand types, smallest movements being recorded in beech forest stands and largest movement distances being recorded in non-spruce conifer and spruce-mixed forest stands, explaining 16% of the finite-sample variation in observed movement distances (Fig. 3, see Appendix S2 for all posterior values). Movement distances varied with stand age, notably with an evident effects of stand age only being found in beech forest stands: movement distances increased with stand age in beech stands but not in other stand types (Fig. 3). In contrast, the effects of point-level tree assemblages were all low, and we did not find any association with variation in movement distances. Movement distances increased further with daytime in spruce and beech-mixed forest stands, but this effect was less obvious in other stand types due to uncertainty in coefficient estimates (Fig. 3). Movement distance did not reveal any relationship to temperature. Variation in movement among bird individuals accounted for 5% of the finite-sample variation in movement distances but we found no relationship between individual average movement distances and wing size or body mass (results not shown in detail). Posterior modes for autoregression coefficients *α* were for most birds 0 < *α* < 0.5 and for one bird individual 0.5 < *α* < 1, suggesting that movement distances from previous time intervals were only of minor impact (Appendix S2).

### Ranging patterns

**Figure 4:**
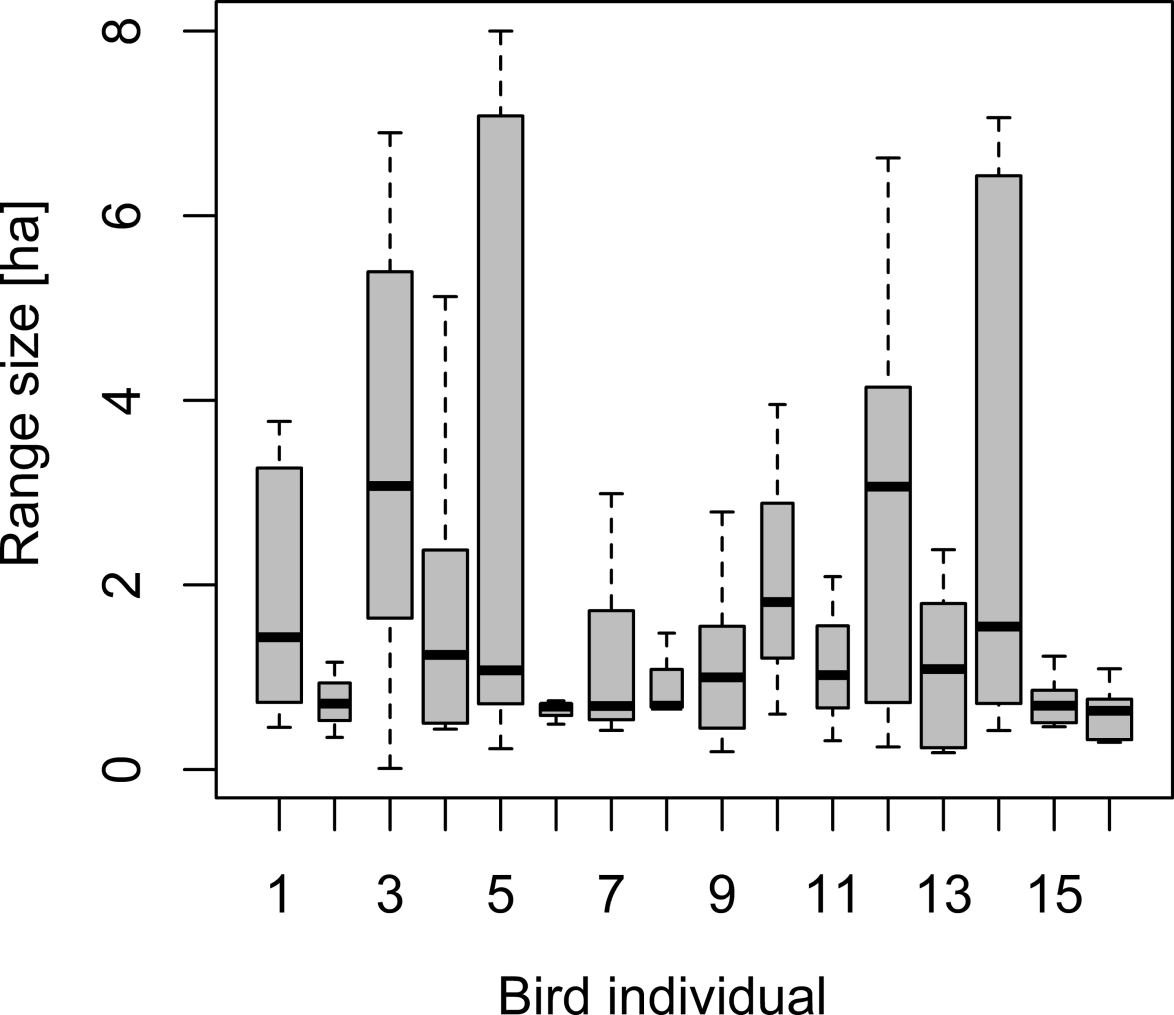
Box–Whisker plot of daily range sizes estimated from 30 locations as 90% isopleths of convex hull polygons. Note that, for individuals 2, 6, 8, 10 and 11 (slim bars), we recorded ranging patterns only for three days, whereas for the other birds, we recorded data for six days. Individuals 3 and 4 refer to range estimates from the same individuals tracked in two consecutive years.

Daily range sizes based on 90% isopleths of convex hulls ranged between 0.01 and 8.0 hectare (median of 0.86 ha), exhibiting a similar magnitude of intra-individual variation over time than variation among individuals foraging in the different forest types (Fig. 4). Indeed, 40% of the variation in daily range size was accounted for by intra-individual variation. None of the covariates characterizing underlying forest cover derived from point-level tree assemblages, regional forest maps, temperature or Julian day revealed any relationship with daily range sizes based on mixed effect models.

Notably, 70% (57 out of 81) of daily range estimates covered more than one forest stand type and also relatively small daily ranges were observed to cover more than three different stand types.

## Discussion

During the tracking of a generalist passerine bird in heterogeneous forest production landscapes, we found that most birds used several different forest stands during their daily movement. Movement distances differed among forest stand types but, in contrast to our expectations, we found little impact of point-level tree assemblages on movement.

Remarkably, stand age and daytime affected movement distance with contrasting effects among stand types, emphasizing that only if we translated the hierarchical organization of forest attributes from point to stand level in a corresponding multilevel hierarchical model could we accurately estimate such effects. Daily ranges exhibited considerable intra-individual variation and also variation among birds but revealed no relationship with the underlying forest, temperature or Julian day. Overall, fine-scale differences in forest management appeared to have less impact on the movement and ranging of chaffinches than areal forest attributes at the stand and landscape level—at least for the chaffinch as a habitat generalist. However, we emphasize that results might both depend on the underlying habitat heterogeneity studied, the way that this is considered for the analysis of different movement trajectories, and also the sensitivity of focal species to habitat changes. Generally, we can expect the combination of environmental and individual conditions, beside other factors, to result in spatiotemporal variable movement trajectories and range dynamics (Dalziel, Morales & Fryxell, 2008). In particular, the considerable intra-individual variation in movement and ranging of birds residing within the same environment suggests that environmental conditions are not the only drivers of variation in movement behaviour in our study. Individual behaviour typical for chaffinches, such territorial defence and even extra-pair mating forays that require movements outside the core territory, for example, suggest that social behaviour might drive movement and ranging of chaffinches as much as foraging decisions linked to the environment (Cherenkov, 2011). In turn, as most birds used various forest stands during their daily movement in our study, we can expect individual birds to experience considerably fewer environmental contrasts than if we had compared birds from highly distinct forests. Fine-scale forest heterogeneity might thus blur any differences in movement behaviour driven by particular environmental conditions and might explain why we found little predictive power of forest attributes for daily range sizes.

We conceptualized the hierarchical organization of small-scale forest heterogeneity from point- to stand-scale into a multilevel model that allowed for decomposing the different sources of variation, while representing underlying forest heterogeneity in a consistent framework. Such multilevel models add to the notion that potential drivers of ranging patterns can only be understood if the variation in ranges is decomposed in spatiotemporal and individual-level processes (Börger et al., 2006; Kie et al., 2010). In particular, we emphasize that forest heterogeneity such as in our study might necessitate the consideration of forest attributes such as forest stand types as group-level predictors but also the grouping of indicators for finer-scale attributes nested within the different tree stands. Analytical multilevel frameworks are well outlined in the literature (Gelman & Hill, 2007). The necessity for such models was, in our study, particularly evident for the effects of stand age and daytime on movement distances: their contrasting effects on movement distances in the various forest stands would not only go undetected when assuming a constant effect for these covariates but would eventually suggest no effect at all or other misleading trends (results not shown in detail).

Contrary to our initial hypothesis, we found stand-level effects of stand type and stand age to be considerably stronger than the effects of local tree assemblages on movement distances, although local tree assemblages should largely define resource availability and also the conditions for birds to move quickly through the canopy. Larger tree diversity or favourable tree species such as oak, *Querqus robur*, that harbour more diverse arthropod assemblages (Sobek et al., 2009), might impact the local foraging behaviour of chaffinches and other bird species (Böhm & Kalko, 2009). Larger tree density and tree diameters have also been found to impact the foraging of woodpeckers, *Picoides arcticus*, in North American forests (Dudley, Saab & Hollenbeck, 2012). Likewise, canopy structure changes with local tree composition and density and should impact the movement conditions for birds and bats during flight (Jung et al., 2012; Müller, Stadler & Brandl, 2010). More exposed foraging sites through more open canopy might also impact predation risk and consequent time allocation to foraging in open versus more sheltered space (Jones, Krebs & Whittingham, 2006; Whittingham et al., 2004). Unfortunately, a comprehensive assessment of forest structure was not possible during field work, as this is unfeasible for a large number of points, and detailed remote sensing data were not available for all point locations. Our study is preliminary in that we have only studied a habitat generalist and lack comparative data for specialist species, which can be expected to be more limited in their movement behaviour and distribution in heterogeneous forest landscapes and are often of particular conservational concern (Dudley, Saab & Hollenbeck, 2012). We nevertheless emphasize that taking the hierarchical organisation of forest structure and attributes into account will advance our understanding how birds or other mobile species may adapt to heterogeneous forest environments and whether stand-level forest management or point-level tree assemblages are of more importance for movement behaviour and distribution of species. In practice, future studies that seek to study movement behaviour in relation to hierarchical forest structure would benefit from larger sample sizes, which can be more easily collected for larger animals that allow automatic recording of geographic positions. It would be also desirable to account for different behaviours such as sexual versus foraging motivated movement and territoriality as a step to ultimately link movement to survival and population growth.

In summary, our results show that diverse forest stand types impact the movement patterns of chaffinches but are all likely to provide foraging opportunities, since chaffinches frequently move between the different forest stands. We argued that forest stand characteristics such as stand type and stand age possibly impact movement patterns in combination and that the hierarchical structure of forest attributes necessitates the application of corresponding multilevel analytical frameworks. Our study is only a first step towards management involvement, since equivalent studies are desirable on more specialized bird species for which we expect a larger impact on local tree assemblages and also in study areas with a different forest landscape composition that might impact the overall environmental contrasts that individual birds experience. Possible beneficial conservation efforts in production forests include less intensive set-aside schemes that leave entire patches unmanaged or the conservation of local key structures such as large old trees, standing dead wood or mixed tree assemblages. If economic pressure does not allow broad-scale conservation efforts or if we need better quantitative measurements to argue for more conservation, we expect that further studies on animal-habitat associations, allowing the quantification of point-level to forest stand and landscape scale effects in a consistent multilevel framework, will be pivotal for science-based management decisions in forest and wildlife conservation.

## Supplemental Information

10.7717/peerj.368/supp-1 Appendix S1 Model code for analysing potential relationships between movement rate and hierarchically structured environmental covariates

10.7717/peerj.368/supp-2 Appendix S2 Model output of the hierarchical model of movement rates, given as highest posterior density modes and 95% credible intervals. For nested point-level parameters, estimates are given for the various forest stands

## Additional Information and Declarations

### Data Deposition

The following information was supplied regarding the deposition of related data: https://exploratories.bgc-jena.mpg.de:444/Login/Account.aspx.
