# Supplementary material for: Movement and ranging patterns of the Common Chaffinch in heterogeneous forest landscapes
Source: PeerJ. 2014 Jun 19;2:e368. doi: 10.7717/peerj.368 (PMC4081153; doi:10.7717/peerj.368)
Supplement: Appendix S1 [file peerj-02-368-s001.docx]

# Appendix A1

Movement and ranging patterns of the common chaffinch, *Fringilla coelebs,* in heterogeneous forest landscapes

By Katrin Kubiczek, Swen C. Renner, Stefan M. Böhm, Elisabeth K.V. Kalko, Konstans Wells

Description: Model code for analysing potential relationships between movement rates and hierarchically structured environmental covariates. The model uses BUGS language such as for the freely available OpenBUGS software, which can be found at <http://openbugs.info/w/>

See below the model code for further comments.

########### Start of BUGS model code ##############

model

{

for(b in 1:nbird){

for(d in 1:nday[b]){

move.dist[b,d,1] ~ dnorm(0, 0.01)

for(s in 1:nstep[b,d]){

## Note that data (observed movement distances) are on log-scale

obs.dist[b,d,s] ~ dnorm(move.dist[b,d,s], tau.dist)

}

for(s in 2:nstep[b,d]){

move.dist[b,d,s] <- intercept + mu.bird[b]

+ alpha[b, standtype.S[b,d,s]] + obs.dist_previous[b,d,s]

+ beta[1, standtype.S[b,d,s]] * shannon.P[b,d,s]

+ beta[2, standtype.S[b,d,s]] * beech.P[b,d,s]

+ beta[3, standtype.S[b,d,s]] * spruce.P[b,d,s]

+ beta[4, standtype.S[b,d,s]] * treedensity.P[b,d,s]

+ beta[5, standtype.S[b,d,s]] * temp.P[b,d,s]

+ beta[6, standtype.S[b,d,s]] * time.P[b,d,s]

+ beta[7, standtype.S[b,d,s]] * standage.S[b,d,s]

+ bstand[standtype.S[b,d,s]]

}

}

}

# Imputing missing data in covariates

for(b in 1:nbird){

for(d in 1:nday[b]){

for(s in 1:nstep[b,d]){

standtype.S[b,d,s] ~ dcat(p.standtype[])

obs.dist_previous[b,d,s] ~ dnorm(0, 0.01)

standage.S[b,d,s] ~ dnorm(0, 0.01)

temp.P[b,d,s] ~ dnorm(0, 0.01)

time.P[b,d,s] ~ dnorm(0, 0.01)

shannon.P[b,d,s] ~ dnorm(0, 0.01)

beech.P[b,d,s] ~ dnorm(0, 0.01)

spruce.P[b,d,s] ~ dnorm(0, 0.01)

treedensity.P[b,d,s] ~ dnorm(0, 0.01)

}

}

}

for(i in 1:nstandtype){

p.standtype[i] <- 1/nstandtype

}

## Priors for coefficient estimates

intercept ~ dnorm(0, 0.01)

for(b in 1:nbird){

mu.bird[b] ~ dnorm(0, tau.bird)

mu.bird.Star[b] <- mu.bird[b] – mean(mu.bird[])

alpha[b] ~ dnorm(0, tau.alpha)

}

tau.bird <- pow(sd.bird, -2); sd.bird ~ dunif(0,10)

tau.alpha <- pow(sd.alpha, -2); sd.alpha ~ dunif(0,10)

for(j in 1:7){

for(s in 1:nstandtype){

beta[j, s] ~ dnorm(0, tau.beta[j])

}

tau.beta[j] <- pow(sd.beta[j], -2); sd.beta[j] ~ dunif(0,10)

}

for(s in 1:nstandtype){

bstand[s] ~ dnorm(0, tau.stand)

bstand.Star[s] <- bstand[s] - mean(bstand[])

}

tau.stand <- pow(sd.stand, -2); sd.stand ~ dunif(0,10)

tau.dist <- pow(sd.dist, -2); sd.dist ~ dunif(0,10)

## Calculate residuals for further analysis

for(b in 1:nbird){

for(d in 1:nday[b]){

for(s in 1:nstep[b,d]){

e.y[b,d,s] <- Obs.log.step[b,d,s] - move.dist[b,d,s]

}

}

}

}

########### End of BUGS model code ##############

Note that for the group-level predictors ‘bird identity’ and ‘forest stand type’ (*S.type^S^*, see Table 1 in manuscript), we rescaled estimates for each level based on the mean as

mu.bird.Star[b] <- mu.bird[b] – mean(mu.bird[])

and

bstand.Star[s] <- bstand[s] - mean(bstand[])

These quantities are then well defined for posterior estimation of their effects and speed up convergence of the model, whereas “mu.bird[b]” and “bstand[s]” are not independently identified (see Gelman, A. & Hill, J. 2007, section 19.4). Finite sample variance for these group-level predictors can then be simply calculated as var(bstand.Star[]) and var(mu.bird.Star[]), respectively. For continuous predictors, finite sample variance was calculated as the squared coefficient estimates (note that all predictors have been scaled).

Missing values of environmental covariates and the autoregression term *λ(i,d,t-1)* were modelled by randomly imputing missing values with combination of different values during Markov Chain Monte Carlo (MCMC) sampling, so that uncertainty about missing values is well represented in the uncertainty of posterior estimates and inference (Gelman et al. 2005).

# References

Gelman, A., Carlin, J. B., Stern, H. S. and Rubin, D. B. (2005) Bayesian data analysis. 2nd edn. Chapman & Hall/CRC, Boca Raton, FL, USA.

Gelman, A. and Hill, J. 2007. Data analysis using regression and multilevel/hierarchical models. Cambridge Univ. Press, New York, USA.
