# Supplementary material for: Movement and ranging patterns of the Common Chaffinch in heterogeneous forest landscapes
Source: PeerJ. 2014 Jun 19;2:e368. doi: 10.7717/peerj.368 (PMC4081153; doi:10.7717/peerj.368)
Supplement: Appendix S2 [file peerj-02-368-s002.docx]

# Appendix A2

Movement and ranging patterns of the common chaffinch, *Fringilla coelebs,* in heterogeneous forest landscapes,

By Katrin Kubiczek, Swen C. Renner, Stefan M. Böhm, Elisabeth K.V. Kalko, Konstans Wells

Table A2.1 Model output of the hierarchical model of movement rates, gives as highest posterior density modes and 95 % credible intervals. For nested point-level parameters, estimates are given for the various forest stand, indicated in parenthesis as follows: Be – Beech, Be-mi - Beech-mixed, Sp -Spruce, Sp-mi - Spruce- mixed, Conif - Non-spruce conifer. Note that all continuous variables have been scaled to a mean of zero and one SD prior to analysis.

| **Coefficient /Variable** | **Description** | **Mode** | **95 % Credible interval** |
| --- | --- | --- | --- |
| Deviance | Overall model deviance | 134200 | 134191 - 134208 |
| *T^P^* | Temperature | (Be) 0.00  (Be-mi) 0.00  (Sp) 0.00  (Sp-mi) 0.00  (Conif) 0.00 | -0.22 - 0.23  -0.19 - 0.18  -0.33 - 0.19  -0.34 - 0.28  -0.26 - 0.07 |
| *Dt^P^* | Daytime | (Be) 0.04  (Be-mi) 0.20  (Sp) 0.26  (Sp-mi) 0.08  (Conif) 0.09 | -0.18 - 0.28  0.02 - 0.39  0.11 - 0.44  -0.18 - 0.52  -0.29 - 0.51 |
| *S.age^S^* |  | (Be) 1.28  (Be-mi) -0.09  (Sp) -0.01  (Sp-mi) -0.05  (Conif) -0.02 | 0.88 - 1.73  -0.44 - 0.13  -0.16 - 0.19  -0.51 - 0.24  -0.44 - 0.34 |
| *H^P^* | Tree diversity at point-level | (Be) 0.00  (Be-mi) 0.00  (Sp) 0.00  (Sp-mi) 0.00  (Conif) 0.00 | -0.31 - 0.10  -0.16 - 0.17  -0.10 - 0.19  -0.18 - 0.21  -0.19 - 0.29 |
| *B^P^* | Beech trees around tracking locations | (Be) 0.01  (Be-mi) 0.00  (Sp) 0.01  (Sp-mi) 0.00  (Conif) 0.00 | -0.12 - 0.56  -0.12 - 0.22  -0.13 - 0.43  -0.42 - 0.26  -0.38 - 0.45 |
| *S^P^* | Spruce trees around tracking locations | (Be) 0.00  (Be-mi) 0.00  (Sp) 0.00  (Sp-mi) 0.00  (Conif) 0.00 | -0.33 - 0.59  -0.41 - 0.32  -0.47 - 0.21  -0.32 - 0.56  -0.51 - 0.59 |
| *X^P^* | Number of trees around tracking locations | (Be) 0.00  (Be-mi) -0.01  (Sp) 0.00  (Sp-mi) 0.01  (Conif) 0.00 | -0.21 - 0.80  -0.57 - 0.10  -0.31 - 0.32  -0.30 - 0.68  -0.48 - 0.60 |
| *S.type^S^* (rescaled) | Type of forest stand (Be, Be-mi, Sp, Sp-mi, Conif) | -1.70  0.10  0.31  0.67  0.73 | -2.29 - -1.04  -0.31 - 0.53  -0.07 - 0.76  0.16 - 1.05  -0.18 - 1.45 |
| *α* | Coefficient for 1^st^ order autoregression term (preceding movement distance), allowed to vary among 16 bird individuals | 0.25  0.96  -0.02  0.17  0.01  0.39  0.28  0.10  0.29  0.01  0.05  -0.01  0.06  0.22  0.20  0.06 | 0.13 0.37  0.49 1.38  -0.14 0.12  -0.14 0.33  -0.13 0.14  0.09 0.63  0.04 0.51  -0.25 0.54  0.12 0.50  -0.10 0.13  -0.12 0.26  -0.12 0.10  -0.03 0.15  0.01 0.54  -0.18 0.40  -0.10 0.21 |

Figure A2.1. Posterior coefficient estimates for the autoregression term α in movement distances from 10-min intervals for 15 tracked bird individuals (note that 3^rd^ and 4^th^ estimates from bottom are from the same individuals tracked in two years).

Credible intervals are drawn as grey bars for 50 % and black bars for 95 % intervals.
